# Goethite Reduction by a Neutrophilic Member of the Alphaproteobacterial Genus *Telmatospirillum*

**DOI:** 10.3389/fmicb.2019.02938

**Published:** 2019-12-20

**Authors:** Emma J. Gagen, Julian Zaugg, Gene W. Tyson, Gordon Southam

**Affiliations:** ^1^School of Earth and Environmental Sciences, The University of Queensland, St. Lucia, QLD, Australia; ^2^Australian Centre for Ecogenomics, School of Chemistry and Molecular Biosciences, The University of Queensland, St. Lucia, QLD, Australia

**Keywords:** canga, geomicrobiology, iron cycling, iron duricrusts, *Telmatospirillum*

## Abstract

In tropical iron ore regions, biologically mediated reduction of crystalline iron oxides drives ongoing iron cycling that contributes to the stability of surface duricrusts. This represents a biotechnological opportunity with respect to post-mining rehabilitation attempts, requiring re-formation of these duricrusts. However, cultivated dissimilatory iron reducing bacteria typically reduce crystalline iron oxides quite poorly. A glucose-fermenting microbial consortium capable of reducing at least 27 mmol/L goethite was enriched from an iron duricrust region. Metagenome analysis led to the recovery of a metagenome assembled genome (MAG) of an iron reducer belonging to the alphaproteobacterial genus *Telmatospirillum*. This is the first report of iron reduction within the *Telmatospirillum* and the first reported genome of an iron-reducing, neutrophilic member of the Alphaproteobacteria. The *Telmatospirillum* MAG encodes putative metal transfer reductases (MtrA, MtrB) and a novel, multi-heme outer membrane cytochrome for extracellular electron transfer. In the presence of goethite, short chain fatty acid production shifted significantly in favor of acetate rather than propionate, indicating goethite is a hydrogen sink in the culture. Therefore, the presence of fermentative bacteria likely promotes iron reduction via hydrogen production. Stimulating microbial fermentation has potential to drive reduction of crystalline iron oxides, the rate limiting step for iron duricrust re-formation.

## Introduction

Microbial iron reduction was discovered in the late 1800s (Allison and Scarseth, 1942). Microorganisms capable of coupling the complete oxidation of organic compounds (or hydrogen) to reduction of iron oxides are known as dissimilatory iron reducers. They occur in a wide variety of natural ecosystems including sediments, soils, and groundwater ecosystems, and are significant contributors to global biogeochemical iron cycling. Dissimilatory iron reducing bacteria reduce crystalline iron oxides (goethite, hematite, and magnetite) quite poorly even with excess electron donor, typically reducing <5% of the total iron available (0.64 mM from 14 mM goethite; Lentini et al., 2012), though up to 12.6% (∼60 mM from 500 mM goethite) has been reported for high surface area goethite (Roden and Zachara, 1996). Lentini et al. (2012) proposed that fermentative processes are likely more significant than dissimilatory processes for microbial reduction of crystalline iron oxides. Consistent with this hypothesis, fermentative bacteria have been implicated

in the dissolution of iron oxides that lead to cave formation in iron ore regions dominated by crystalline iron oxides (Parker et al., 2017). In contrast, the dissimilatory iron reducer *Shewanella oneidensis* reduced the iron oxides in that system quite poorly, reducing <2% of total Fe (∼0.62 mM from 40 mM total Fe) without an external electron shuttle provided (Parker et al., 2013).

**FIGURE 1:**
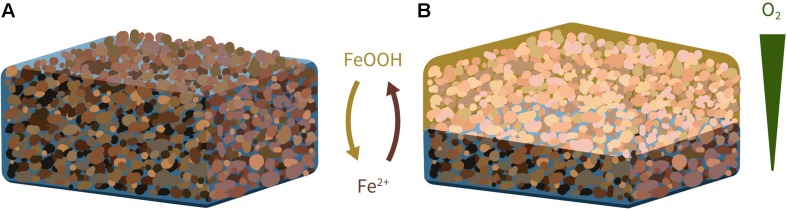
Conceptual model of microbial iron cycling for cementation of crushed canga in a post-mining context. Provision of an abundant and appropriate carbon source will promote effective reduction of crystalline iron oxides in a saturated pile of crushed canga, e.g., during wet season **(A)**, while evaporation, e.g., during the subsequent dry season, will drive oxidation of the ferrous iron rich fluids as the oxidation front progresses downward **(B)**. Neutrophilic iron-oxidizing bacteria will likely play a role at the redox front (Roden et al., 2004).

A hard, goethite-cemented duricrust, known as canga, caps high grade iron ore deposits that formed from the weathering of banded iron formations in tropical regions (Dorr, 1964; Monteiro et al., 2014; Gagen et al., 2019). These geologic features are extensive (kilometer scale) and erosion resistant, forming ridges and plateaus that define the regional landscape. They also underpin a unique plant ecosystem that is a post-mining rehabilitation priority (Gagen et al., 2019). Ongoing biologically mediated iron reduction and subsequent reprecipitation in the goethite cements of canga through geologic time contributes to the evolution and stability of these duricrusts (Monteiro et al., 2014; Gagen et al., 2019). Harnessing these processes in the present day offers an opportunity for post-mining, iron duricrust re-formation during iron mine site rehabilitation. Canga is typically a waste product from iron ore mining and the substrate used for remediation of these sites. Potentially, crushed canga could be re-cemented to form a stable surface crust during site remediation by promoting microbial iron reduction of the material (e.g., in a saturated pile of canga, or in a bioreactor) and allowing subsequent oxidation of the ferrous iron produced (e.g., by dehydration of the saturated pile, or by spraying bioreactor fluids onto other crushed canga areas) and the formation of new iron oxide cements (Figure 1). However, the rate limiting step for the success of an approach like this is reduction of the crystalline iron oxides in canga. In the present study, we enriched an effective fermentation-driven, goethite-reducing microbial consortium from a canga ecosystem, demonstrating the potential for progress in applied biotechnologies that could be used for canga re-formation. An interrogation of the metagenome of the enrichment culture revealed genes encoding metal transfer reductases and novel multi-heme outer membrane cytochromes, in a *Telmatospirillum*. This represents the first report of iron reduction within the *Telmatospirillum* and extends the diversity of iron reduction capabilities to include neutrophilic members of the Alphaproteobacteria.

## Materials and Methods

### Sampling

Samples from freshly forming iron oxides, pools of water perched on iron duricrust and the gut of termites that build their mounds in and on the iron duricrust in the iron ore regions of Western Australia and Brazil, were collected into sterile containers in the field and stored at 4°C before later cultivation attempts.

### Enrichment Cultures

The base medium used to enrich iron reducers was prepared anaerobically, and contained per liter: 2.5 g NaHCO_3_, 1.5 g NH_4_Cl, 0.68 g KH_2_PO_4_, 0.1 g KCl and was pH adjusted to pH ∼6.8. Iron was usually provided as goethite powder (0.5 g/100 ml or 0.1 g/10 ml) or as crushed canga (1 g/10 ml), added to serum vials or Hungate tubes individually before flushing with nitrogen and adding liquid in an anaerobic chamber of gas phase N_2_:H_2_ (95:5). Prior to microbial inoculation, electron donors were added from sterile anaerobic stock solutions to final concentrations of 30 mM sodium lactate, 30 mM sodium acetate, and/or 10 mM glucose unless otherwise indicated. Stable enrichment cultures were routinely grown on the base medium with goethite (1.6 g/80 ml; ∼225 mmol/L) and 30 mM glucose in 160 ml serum vials. Culture characterization was performed in triplicate cultures, after the 5th transfer from the original inoculum. Cultures were routinely transferred after plateau of ferrous iron production (any time after 2 weeks since inoculation).

### Chemical Analyses

Cultures were monitored for the production of ferrous iron spectrophotometrically using the ferrozine assay modified from Lovley and Phillips (1986). To measure total ferrous iron, a sample of culture (0.1 ml) was mixed with 5 ml of 0.5 N HCl for 15 min, then 0.2 ml of this mixture was added to 2 ml ferrozine (1 g/L in 50 mM HEPES) and filtered through a 0.2-μm pore size filter. To determine soluble ferrous iron, the procedure was the same except that a sample of culture was filtered through a 0.2-μm pore size filter before 0.1 ml of the filtrate was acidified and mixed using ferrozine. The absorbance was measured at 562 nm and compared to known standard concentrations of Fe(II) prepared anaerobically ferrous chloride.

A Shimadzu Prominence HPLC system (Kyoto, Japan) was used to determine the presence of formic, lactic, acetic, butyric, propionic, succinic, isovaleric, and *n*-valeric acids and glucose in culture supernatant. Separation and identification of acids from 20 μl sample was achieved using an Agilent Hi-Plex H Column (8 μm, 7.7 × 300 mm, Agilent Technologies, Australia) at 35°C using a mobile phase of 14 mM H_2_SO_4_ at 0.5 ml/min.

### DNA Extraction and Amplicon Sequencing

For cultures, 0.5 ml of each of three late log phase cultures was centrifuged (13,000 × *g* × 5 min) for DNA extraction using the DNEasy Powersoil kit (Qiagen, Singapore) as per manufacturer’s instructions. For the water sample from which the cultures originated, 40 ml of water had been collected onto a Sterivex filter in the field and the filter was cut into pieces for DNA extraction using the same kit. The V6–V8 region of the 16S rRNA gene was amplified using primers 926F and 1392r and sequenced on a MiSeq Sequencing System (Illumina, San Diego, CA, United States) [see Gagen et al. (2018) for primer sequences, library preparation, and sequence analysis using MOTHUR (Schloss et al., 2009) and the Silva reference database SSU Ref NR 99 v132 (Quast et al., 2012; Yilmaz et al., 2013)]. A heatmap was generated to visualize dominant OTUs in the samples using the package ComplexHeatmap (ver 2.1.0) in R (ver. 3.5.2) (Gu et al., 2016). Sequences have been submitted to GenBank Sequence Read Archive under BioProject number PRNJA561022.

### Metagenome Library Preparation and Sequencing

DNA concentration was measured using a Qubit 3.0 high sensitivity assay (ThermoFisher Scientific, Waltham, MA, United States) and was adjusted to 5 ng/μl. A metagenome library was prepared using Nextera XT Library Preparation Kit (Illumina, San Diego, CA, United States, #FC-131-1096) according to the manufacturer’s protocol except adjusting the reaction volume to allow for processing in 96-well plate format. Library preparation and bead clean-up were performed using the Mantis Liquid Handler (Formulatrix, Bedford, MA, United States) and Epmotion (Eppendorf, Hamburg, Germany, #5075000301) automated platform. These programs cover “Tagment Genomic DNA” to “Amplify DNA” in the protocol (Mantis – Nextera XT library prep protocol) and “Clean Up Libraries” in the protocol (Epmotion – Library Clean Up protocol). On completion of the library prep protocol, the library was quantified and quality control was performed using the Quant-iT^TM^ dsDNA HS assay kit (Invitrogen, Carlsbad, CA, United States) and Agilent D1000 HS tapes (Agilent, Santa Clara, CA, United States, #5067-5582) on the TapeStation 4200 (Agilent, Santa Clara, CA, United States, #G2991AA) as per manufacturer’s protocol. The library was pooled with other libraries at equimolar amounts of 2 nM per library, to create a sequencing pool. The library pool was quantified in triplicate using the Qubit^TM^ dsDNA HS Assay Kit (Invitrogen, Carlsbad, CA, United States). Library QC was performed using the Agilent D1000 HS tapes on the TapeStation 4200 as previously. The library was prepared for sequencing on the NextSeq500 (Illumina, San Diego, CA, United States) using NextSeq 500/550 High Output v2 2 × 150 bp paired end chemistry, at the Australian Centre for Ecogenomics, according to manufacturer’s protocol.

### Genome Assembly

Raw reads were processed with Trimmomatic (Bolger et al., 2014) (ver. 0.36; ILLUMINACLIP:NexteraPE-PE.fa:2:30:10, LEADING:3, TRAILING:3, SLIDINGWINDOW:4:15, HEADCROP:0, MINLEN:50) for adapter removal and quality filtering. Reads were then assembled using MetaSPAdes (Nurk et al., 2017) (ver. 3.11.1) with default parameters. Contigs whose length was <500 bp were removed using BBMap^1^ (ver. 38.41). Raw and assembled reads have been submitted to GenBank Sequence Read Archive under BioProject number PRNJA561022.

### Binning, Dereplication, and Taxonomic Classification

Quality controlled reads for each sample were mapped onto their respective assemblies using CoverM “make”^2^ (ver 0.2.0, B. Woodcroft, unpublished). Low quality mappings were removed with CoverM “filter” (minimum identity of 95% and minimum aligned length of 75%). Assemblies for each sample were binned by providing the contigs for each sample and filtered BAM files as input to UniteM^3^ (ver. 0.0.15, D. Parks, unpublished) and using a minimum contig length of 1,500 bp and Maxbin (ver. 2.2.4), MetaBAT (ver. 0.32.5), and MetaBAT2 (ver. 2.12.1) binning methods (max40, max107, mb2, mb_verysensitive, mb_sensitive, mb_specific, mb_veryspecific, and mb_superspecific). Bins were dereplicated using dRep (Olm et al., 2017) (ver. 2.2.3, sa = 0.95, comp = 40, con = 15). Bin completeness and contamination were evaluated using CheckM (Parks et al., 2015) (ver. 1.0.12). Taxonomies were assigned to each bin using GTDB-tk^4^ (Parks et al., 2018) (ver. 0.3.0).

### Calculation of Bin/Metagenome Assembled Genome Abundances

To calculate the relative abundance of each bin, reads from each sample were mapped and filtered as was done for the unbinned assemblies. After filtering, 80% of the reads mapped to the bins. The mean coverage of each bin was calculated with CoverM. The relative abundance of each bin, among those recovered, was calculated as its coverage divided by the total summed coverage of all bins. Abundance values were multiplied by the fraction of reads that mapped to all bins after filtering to produce the relative abundance of each bin within the entire sample.

### Gene Extraction and Functional Annotation

Bin assemblies were translated and functionally annotated using a combination of Prokka (Seemann, 2014) (ver 1.13.3), Prodigal (Hyatt et al., 2010) (ver. 2.6.3), and EnrichM^5^ (ver. 0.4.15, J. Boyd, in preparation), the latter using annotation options –ko_hmm, –pfam, –tigrfam, –orthologs, –clusters, –cazy, and –ec. In EnrichM, for a query gene to be considered for annotation, the minimum fraction aligning to a reference, and *vice versa*, was set to 0.5, with a minimum percent identity of 30% also required.

### Cytochromes, Hydrogenases, and Enzymes Involved in Iron Reduction

Potential *c*-type cytochromes were identified by determining encoded proteins with heme-binding motifs. For each bin, heme-binding motifs reported in the literature (Ferguson et al., 2008; Onley et al., 2018) were identified in all translated coding genes (amino acid sequences) outputted by Prokka, through pattern matching and counting. Hydrogenases were grouped by type as per Greening et al. (2015). Genes encoding proteins important in iron reduction in *S. oneidensis* (CctA, CymA, FccA, MtrA, MtrB, MtrC, MtrD, MtrE, MtrF, MshA, MshB, MshC, MshD, OmcA, and PilA) *Geobacter sulfurreducens* (ImcH, OmcB, OmcC, OmcE, OmcS, and PilA), and *Desulfuromonas acetoxidans* (cytochrome c7) were determined by BLAST analysis of the publicly available (UniProt) amino acid sequences against deduced amino acid sequences from contigs from the metagenome. Matches were considered significant (i.e., worth further investigation) at *e*-values <1*e*^–20^.

## Results

### Optimizing Carbon Substrate for Goethite Reduction From Canga Ecosystems

Goethite and canga-reducing cultures prepared from various iron duricrust-associated samples collected in Western Australia and Brazil were initially enriched using a mixture of lactate, acetate, and glucose as per Lentini et al. (2012). When electron donors were examined individually, the cultures were found to produce equivalent ferrous iron on glucose alone, as they did on the mixture of carbon sources. Compared to when the electron donor was lactate alone, glucose-oxidizing cultures produced maximum ferrous iron much faster (6–12 days compared to 40–70 days) and to higher concentration (e.g., ∼15 mM compared to ∼5 mM, data not shown). In light of these preliminary observations from a range of cultures, and given the biotechnological potential for an effective goethite-reducing microbial culture, subsequent enrichments were prepared by inoculating freshly collected samples from iron ore regions, onto goethite in a sulfate-free basal medium, with glucose (30 mM) as the electron donor. The culture found to reduce the most goethite was characterized in more detail and will be referred to as GluFeRB. It had been enriched from a pool perched on canga (see the section “Microbial Diversity in Iron Duricrust-Associated Sample From Which GluFeRB Was Obtained”) and was passaged five times before detailed culture characterization and DNA-based analyses (sections “Characterization of Culture GluFeRB,” “16S rRNA Gene Data From Glucose-Oxidizing, Goethite-Reducing Culture GluFeRB on Sulfate-Free Medium,” and “Metagenomic Analysis of Glucose-Oxidizing Goethite Reducing Enrichment Culture on Sulfate-Free Medium”).

### Microbial Diversity in Iron Duricrust-Associated Sample From Which GluFeRB Was Obtained

GluFeRB was enriched from water collected from a small pool perched on canga near Lake Violão, Carajás, Brazil (S06°24′07.5″ W050°21′05.6″). Ferrous iron was detected in the pool (purple color reaction of ferrozine assay) in the field, March 2016. Bacterial and archaeal 16S rRNA genes in the environmental sample clustered into 3960 OTUs (at <0.03% distance). Rarefaction analysis indicated that further microbial diversity would be recovered with deeper sequencing (data not shown). The microbial community was dominated by lineages that typically contain anaerobes (e.g., Methanobacteriaceae, Veillonellaceae, Clostridiaceae, Ruminococcaceae, Methanospirillaceae, Supplementary Figure 1) suggesting anaerobic fermentation and methanogenesis were major processes in the pool perched on canga. However, sequences from a phototrophic oxygenic lineage were also abundant (OTU 16, Supplementary Figure 1, 1.6% of sequences) and a *Curvibacter* (OTU 22, Supplementary Figure 1), a lineage recently implicated in neutrophilic iron oxidation (Gülay et al., 2018) was also abundant (1.17% of sequences). Known iron-reducing lineages were not abundant in the community profile. An *Anaeromyxobacter* OTU was the most abundant known iron-reducing microbial lineage detected, at 0.1% of the total sequences. A *Geobacter* OTU was also detected at 0.06% of total sequences. An OTU present at 0.55% (OTU 39, Supplementary Figure 1) was most similar to *Azonexus* WE2-4 which has been shown to be capable of extracellular electron transfer under anaerobic conditions (Jangir et al., 2016) and potentially could be contributing to iron cycling in the natural ecosystem.

### Characterization of Culture GluFeRB

**FIGURE 2:**
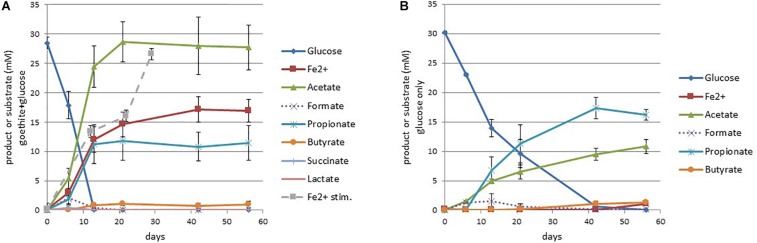
Short chain fatty acid and ferrous iron production with glucose consumption for cultures with **(A)** and without **(B)** goethite. The dashed line (Fe^2+^ stimulation in graph **A**) is measured ferrous iron when cultures were provided with more glucose (30 mM) at day 22. Error bars represent the standard error of the mean (*n* = 3).

In the presence of goethite (1 g/50 ml = 225 mM), on sulfate-free medium, culture GluFeRB consumed glucose (30 mM) completely within 13 days and produced acetate (27.7 mM), propionate (11.4 mM), ferrous iron (16.9 mM), and a minor amount of butyrate (0.94 mM, Figure 2). A subsample from one of the cultures showed that the pH dropped from ∼6.9 to 6.24 across this period. Acetate and ferrous iron continued to increase slightly after the consumption of glucose (Figure 2). Ferrous iron production could be re-stimulated in GluFeRB cultures that had reached a ferrous iron plateau simply by the addition of more glucose (dashed line, Figure 2A), indicating that ferrous iron toxicity was not responsible for cessation of iron reduction. The proportion of measured ferrous iron that was soluble (i.e., not adsorbed to the goethite) in GluFeRB cultures was found to be 76.3 ± 1.86% (measured at end point, in later transfers of the culture). Formate (2.1 mM), lactate (0.19 mM), and succinate (0.38 mM) were produced in the first 6 days and consumed within the next 7–14 days. In comparison, when GluFeRB was grown on glucose only without goethite, glucose was consumed much slower (<42 days) and the major end product was propionate (16.19 mM) with a smaller amount of acetate (10.8 mM) and minor butyrate (1.3 mM; Figure 2B). Lactate was never detected (Figure 2) and acetate:propionate was much higher in the presence of goethite (Ac:Pr 2.42) compared to cultures not provided with goethite (Ac:Pr 0.67; Figure 2).

### 16S rRNA Gene Data From Glucose-Oxidizing, Goethite-Reducing Culture GluFeRB on Sulfate-Free Medium

GluFeRB was dominated by two OTUs at almost equal proportions, one demonstrating 95% 16S rRNA gene identity across the sequenced region to the nearest named isolate *Elstera litoralis* and the other demonstrating 98% 16S rRNA gene identity to *Paludibacter jiangxiensis*, neither of which are reported to be able to use ferric iron as a terminal electron acceptor (Rahalkar et al., 2012; Qiu et al., 2014). The five next most abundant OTUs indicated the likely presence of a hydrogenotrophic methanogen [100% 16S rRNA gene identity to *Methanobacterium lacus* (Cadillo-Quiroz et al., 2014)], a sulfate reducing bacterium (100% 16S rRNA gene identity to various strains of *Desulfovibrio desulfuricans*), two closely related fermentative bacteria (both OTUs > 99% 16S rRNA gene identity to *Bacteroides xylanolyticum*), and a *Clostridium* species [100% 16S rRNA gene identity to *Clostridium aciditolerans* (Lee et al., 2007)]. The dominant members of culture GluFeRB were not detected in the amplicon data from the parent material and the other members of the culture were also low abundance (*Methanobacterium* 0.67%, *Desulfovibrio* 0.25%, *Clostridium* OTUs 0.56–0.005% of total sequences, Supplementary Figure 1).

### Metagenomic Analysis of Glucose-Oxidizing Goethite Reducing Enrichment Culture on Sulfate-Free Medium

Six metagenome assembled genomes (MAGs) were reconstructed from metagenomic data from culture GluFeRB (Table 1). These represent population genomes for the main organisms (80% of total metagenome reads mapped to these MAGs) in the glucose-oxidizing, goethite-reducing culture on sulfate-free medium. MAGs were taxonomically classified using the Genome Taxonomy Database toolkit (GTDB tk) version 3.0 (see text footnote 4) as falling within the genera *Telmatospirillum*, *Paludibacter*, *Desulfovibrio*, *Hungatella*, *Methanobacterium*, and the family Clostridiaceae (Table 1). Genome phylogeny for these six MAGs was consistent with the most abundant OTUs by 16S rRNA gene analysis, except that the two OTUs most closely related to *B. xyanloyticum* at the 16S rRNA gene level, are likely represented by a single MAG. Relative abundances of the MAGs were consistent with those observed for correspondingly classified OTUs except for *Methanobacterium* which was overrepresented in the 16S rRNA gene data (11.5% of 16S rRNA gene sequences, 3rd most abundant OTU) compared to the metagenomic data (1.0% relative abundance, 6th most abundant genome).

**TABLE 1:** Taxonomic classification and summary statistics for MAGs from culture GluFeRB.

**Relative abundance (%)**	**GTDB taxonomy**	**Nearest reference genome and average nucleotide identity**	**Genome length (bp)**	**Completeness (%)**	**Contamination (%)**	**N50 (contigs)**
30.7	d__Bacteria;p__Proteobacteria;c__Alphaproteobacteria;o__ Rhodospirillales;f__Magnetospirillaceae;g__**Telmatospirillum**;s_	*Telmatospirillum siberiense*, GCF_002845745.1 (78.61%)	6112934	99.5	3.53	41887
29.5	d__Bacteria;p__Bacteroidota;c__Bacteroidia;o__Bacteroidales;f__ Paludibacteraceae;g__**Paludibacter**_A;s__	*Paludibacter jiangxiensis*, GCF_001618385.1 (89.67%)	3695992	97.58	0	527666
8.9	d__Bacteria;p__Desulfobacterota;c__Desulfovibrionia;o__ Desulfovibrionales;f__Desulfovibrionaceae;g__**Desulfovibrio**;s__	*Desulfovibrio desulfuricans*, GCF_000420465.1 (85.01%)	3668018	99.7	0.64	150073
8.7	d__Bacteria;p__Firmicutes_A;c__Clostridia;o__Lachnospirales;f__ Lachnospiraceae;g__**Hungatella**;s__	*Hungatella xylanolyticus*, GCF_002934545.1 (94.63%)	5631814	99.37	5.06	505197
1.0	d__Bacteria;p__Firmicutes_A;c__Clostridia;o__ Clostridiales;f__**Clostridiaceae**;g_;s__	*Clostridium magnum*, GCF_001623875.1 (79.95%)	4224202	84.19	3.18	4598
1.0	d__Archaea;p__Euryarchaeota;c__Methanobacteria;o__ Methanobacteriales;f__Methanobacteriaceae;g__ **Methanobacterium**_B;s__	*Methanobacterium lacus*, GCF_000191585.1 (88.82%)	2181304	94.13	0.8	5684

### Iron Reduction Potential Within the Metagenome From Culture GluFeRB

**FIGURE 3:**
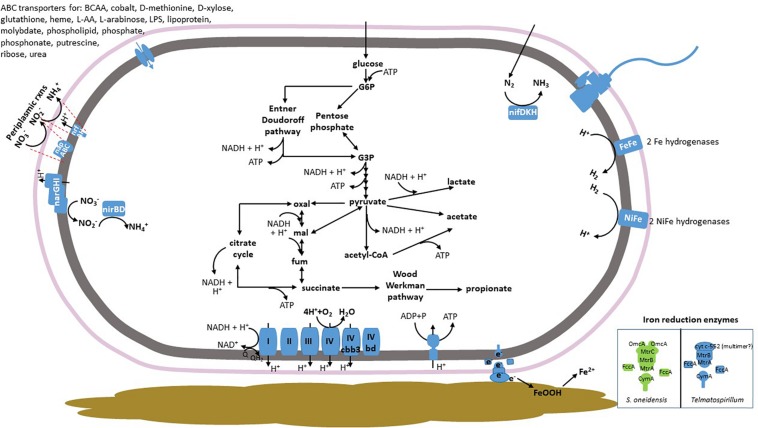
Metabolic potential of the *Telmatospirillum* MAG in culture GluFeRB. Electrons are shown as e^–^ where the exact carrier isn’t known (in the case of iron reduction). Inset in the bottom right-hand corner shows comparison of iron reduction enzymes in *Shewanella oneidensis* (green) and the *Telmatospirillum* MAG. BCAA, branched chain amino acids; L-AA, L-amino acids; LPS, lipopolysaccharide; G6P, glucose-6-phosphate; G3P, glyceraldehyde-3-phosphate: oxal, oxaloacetate; mal, malate; fum, fumarate.

A search for proteins involved in iron cycling, based on those characterized in *S. oneidensis* or *G. sulfurreducens*, revealed putative iron reduction capabilities within the *Telmatospirillum* MAG (Table 2). Two putative MtrA proteins (with 10 and 9 predicted heme-binding motifs, respectively) demonstrated high homology (Table 2) to MtrA in *S. oneidensis* and are likely DmsE family decaheme c-type cytochromes. Both genes were neighbored by genes encoding proteins demonstrating homology to transmembrane porin MtrB/PioB family proteins (Table 2). An extensive search for MtrC and OmcA homologs in the *Telmatospirillum* MAG did not return any significant matches (the genes encoding putative MtrA were the most significant to MtrC at *e*-values ∼1*e*^–7^). However, a cytochrome c-552 family protein with two predicted heme-binding motifs directly neighbors one of the putative MtrA genes (Table 2) and is predicted to be present in the extracellular space based on BUSCA analysis (Savojardo et al., 2018). It is the most likely candidate for extracellular electron transfer from the membrane-spanning MtrAB complex to goethite and/or to electron shuttles (Figure 3).

**TABLE 2:** Enzymes potentially involved in iron cycling, identified in the metagenome of GluFeRB.

**MAG**	**Gene name, Prokka annotation, and predicted number of heme-binding motifs^a^**	**Homolog in *S. oneidensis***	***e*-value**
*Telmatospirillum*	SA7853BIN3_04712 hypothetical protein (10 hemes)	MtrA *S. oneidensis*	1*e*^–71^
*Telmatospirillum*	SA7853BIN3_04496 hypothetical protein (9 hemes)	MtrA *S. oneidensis*	1*e*^–70^
*Telmatospirillum*	SA7853BIN3_04713 hypothetical protein	MtrB *S. oneidensis*	9*e*^–23^
*Telmatospirillum*	SA7853BIN3_04495 hypothetical protein	MtrB *S. oneidensis*	3*e*^–22^
*Telmatospirillum*	SA7853BIN3_04711 cytochrome c-552 (2 hemes)	none	n/a
*Telmatospirillum*	SA7853BIN3_04245 denitrification system component NirT (4 hemes)	CymA in *S. oneidensis*	2*e*^–22^
*Telmatospirillum*	SA7853BIN3_00738 urocanate reductase	FccA in *S. oneidensis*	8*e*^–64^

*^*a*^Amino acid sequences are available in Supplementary Table 1.*

A homolog to CymA, the tetraheme cytoplasmic membrane protein that transfers electrons from the quinol pool to periplasmic proteins [in the case of iron reduction, to the carrier FccA or to MtrA directly (Liu et al., 2018)] is also present in the *Telmatospirillum* MAG (Table 2). It exists within an operon of genes for nitrate reduction, reflective of the role this protein plays in electron transport to various electron acceptors, e.g., iron, fumarate, nitrate, and manganese (Myers and Myers, 2000). The *Telmatospirillum* MAG also encodes a protein demonstrating high homology to FccA in *S. oneidensis* (Table 2). None of the other MAGs in culture GluFeRB contained homologs for characterized outer membrane metal reduction proteins. A search for cytochrome c7 homologs [cyt c7 is involved in metal reduction in some sulfur reducing bacteria (Banci and Assfalg, 2006)] in the metagenome did not return any significant matches.

### Other Respiratory Pathways Within the Metagenome From Culture GluFeRB

Respiratory complexes I–IV are present in *Telmatospirillum* (Figure 3), as well as genes encoding the high-affinity cytochrome cbb3 type and cytochrome bd complex terminal oxidases that are typically expressed under microaerobic conditions (Pitcher and Watmough, 2004; Borisov et al., 2011). The *Paludibacter* and *Desulfovibrio* MAGs also encode respiratory complexes I, II, and cytochrome bd complex (Supplementary Figures 2, 3).

The *Desulfovibrio* and *Telmatospirillum* MAGs demonstrate evidence for nitrate reduction, and the *Desulfovibrio* also encodes the proteins for sulfate reduction (Figure 3 and Supplementary Figure 3). Both *Telmatospirillum* and *Desulfovibrio* MAGs encoded multiple multi-heme (>2 hemes) cytochromes (at least 11 and 14, respectively) further indicative of respiratory flexibility, while multi-heme cytochromes were not predicted in other bacterial MAGs in GluFeRB.

### Other Observations From the Metagenome of Culture GluFeRB

The four most abundant organisms in the culture seemed dependent on heterotrophy (Embden–Meyerhof–Parnas or Entner Doudoroff pathways) for carbon, while the lower abundance Clostridiaceae and *Methanobacterium* MAGs contained genes for carbon fixation via the Wood–Ljungdahl pathway. Genes encoding enzymes involved in mixed acid fermentation were encoded in all bacterial species, with major end products predicted to be acetate, propionate, and lactate (Figure 3 and Supplementary Figures 2, 3). All bacterial MAGs contained both hydrogen-consuming and hydrogen-evolving hydrogenases (Greening et al., 2015) with more FeFe hydrogenases (likely hydrogen evolving) in the predicted fermenters *Hungatella* and Clostridiaceae, as expected. All MAGs were predicted to encode molybdenum nitrogenases for nitrogen fixation and *Methanobacterium* also encoded an iron-containing nitrogenase. *Hungatella*, Clostridiaceae, *Telmatospirillum*, and *Desulfovibrio* are likely motile based on the presence of proton driven flagellar assembly machinery.

## Discussion

Iron reduction is a relatively widespread trait, though microorganisms capable of effectively reducing the crystalline sources of iron oxide are limited. In the present study, we produced an effective goethite-reducing mixed culture, capable of reducing up to 12% of provided goethite with further reduction beyond this likely, given observations that goethite reduction could be stimulated after reaching plateau, simply by addition of more glucose (dashed line, Figure 1A). While <3% of the electrons from glucose were recovered as ferrous iron (reaction 1), the presence of goethite significantly promoted glucose fermentation, i.e., glucose consumption was 3× faster than in the absence of goethite, and goethite was rapidly reduced.

CH6O12+624FeOOH+48H+

(1)  →24Fe+2+42HO2+6CO2

In the iron ore regions of both Brazil and Australia, goethite reduction (and subsequent reprecipitation) is significant and has been ongoing through geologic time, over large scales, contributing to long-term stability of surface duricrusts (Monteiro et al., 2014; Gagen et al., 2018, 2019; Monteiro et al., 2018a, b). In samples from the iron duricrust regions of both Brazil and Australia in this study, glucose served as a much more effective carbon source for goethite reduction, than did lactate or acetate, consistent with previous observations for crystalline iron oxides (Lentini et al., 2012). Furthermore, the marked absence of well-known dissimilatory iron reducers (e.g., *Geobacter* and *Shewanella*) in cultures from both these regions suggests important microbial iron-cycling potential that is currently being overlooked in these systems.

Our data confirm that fermentative processes could be important with respect to iron cycling in iron duricrust regions, as has been suggested based on microbial community profiling (Parker et al., 2017). Furthermore, culture GluFeRB reduced markedly more crystalline iron oxide than the dissimilatory iron reducer *S. oneidensis*, even accounting for the improved energy available from 30 mM glucose compared to 20 mM lactate used by Parker et al. (2013). Presumably the rate-limiting step for fermentation coupled to reduction of crystalline iron oxides in the duricrust regions naturally is the presence of fermentable substrates and anaerobic niches. It is likely that syntrophic relationships between major fermenters (e.g., *Paludibacter*, Supplementary Figure 2) and the predicted metal reducer *Telmatospirillum* (Figure 3) also contribute to the effective goethite reduction in mixed culture GluFeRB, compared to pure culture dissimilatory iron reducers. For example, interspecies hydrogen transfer between community members seems a likely possibility, and consumption/production of specific short chain fatty acids may also have favored goethite reduction, or the activity of the *Telmatospirillum*. None of the organisms enriched for in culture GluFeRB were abundant in the environmental sample, however, our cultures suggest that simply providing an appropriate carbon source can stimulate fermentation and the interspecies interactions that promote reduction of crystalline iron oxides which are abundant in that ecosystem. We have previously noted that overall broad-scale anaerobic conditions and an abundance of carbon source might result in net dissolution (e.g., cave formation) rather than the dissolution and re-precipitation that leads to iron duricrust stabilization (Gagen et al., 2018). In the environmental sample from which GluFeRB was obtained, a putative iron-oxidizing lineage and an oxygenic phototrophic lineage were more abundant (1.6 and 1.2% of total sequences, respectively) than known iron reducing lineages (<0.1% of total sequences), suggesting overall net precipitation of iron oxide *in situ* at the time of sampling.

Initially, in culture GluFeRB we suspected that the *Desulfovibrio* might be responsible for goethite reduction, even though it was present at <10% abundance (16S rRNA genes) on sulfate-free medium. Iron reduction has been reported for members of the *Desulfovibrio* previously, however, has not been coupled to cell growth (Caffrey et al., 2008). In fact, with iron as the only electron acceptor (i.e., sulfate-free medium) *Desulfovibrio vulgaris* cell numbers have been reported to decline (Caffrey et al., 2008). We therefore undertook genome-resolved metagenomics in order to investigate the intriguing mechanism that allowed members of culture GluFeRB to reduce crystalline iron oxides in the absence of sulfate and seemingly to do so with an energetic gain (e.g., compare glucose consumption in Figures 2A, 2B). Surprisingly, metagenomics revealed the capacity for iron reduction within the *Telmatospirillum* MAG. The nearest cultured representative to the *Telmatospirillum* in GluFeRB is *Telmatospirillum siberiense* (78.61% average nucleotide identity across the genome, GCF_002845745.1), a facultative anaerobe capable of fermentative growth (Sizova et al., 2007; Hausmann et al., 2018). Iron reduction has never been reported for this genus, though *Telmatospirillum* sequences have been found in other studies investigating microbial iron cycling (Mühling et al., 2016).

In *S. oneidensis*, MtrA and MtrB form a complex with decaheme cytochromes MtrC and/or OmcA, which are exposed on the outer surface of the outer membrane and transfer electrons to solid electron acceptors or electron shuttling molecules [e.g., see Liu et al. (2018) for a review]. Two copies of genes encoding homologs to MtrA and MtrB were detected in *Telmatospirillum*, while homologs for MtrC and OmcA were not. The absence of MtrC and OmcA homologs in *Telmatospirillum* is not surprising, given the low conservation of cytochromes that transfer electrons to external electron acceptors even within species of the same genus (Butler et al., 2010). We propose that the di-heme cytochrome c protein that exists in an operon with one set of the MtrAB genes is involved in electron transfer to solid surfaces in *Telmatospirillum*. The gene order in this *Telmatospirillum* operon (cytochrome c-552, MtrA, MtrB) is consistent with that for MtrC, MtrA, and MtrB in *S. oneidensis* (MtrCAB). Furthermore, the cytochrome c-552 protein is predicted to exist in the extracellular space, as would be required for transfer of electrons to insoluble electron acceptors such as goethite. Other cytochrome c-552 family proteins exist as functional dimers (Einsle et al., 1999), such that the functional protein complex has 10 heme groups. A multimer may be the case for the cytochrome c552 protein in *Telmatospirillum*, which is predicted to only have 2 hemes in a single unit, while characterized MtrC and OmcA proteins have 10 hemes.

To date there is a dearth of information on the mechanisms of iron reduction in genera other than *Geobacter* and *Shewanella*. *Telmatospirillum* might represent a useful model for understanding iron reduction outside the Gammaproteobacteria. Genome analysis suggests that iron reduction potential is widespread among other members of the *Rhodospirillales* with MtrA, MtrB homologs present in *T. siberiense*, *Nitrosococcus halophilus*, *Skermanella stibiiresistens*, *Insolitispirillum peregrinum*, and *Magnetospirillum magneticum*. Acidophilic Rhodospirillales have been shown to reduce iron under acidic, microaerobic conditions (Coupland and Johnson, 2008). Although the mechanism of iron reduction in those organisms is unpublished there are indications that it also involves a novel outer membrane cytochrome c^6^.

In GluFeRB glucose fermentation in the presence of goethite shifted short chain fatty acid production in favor of acetate rather than propionate (Figure 2). In the absence of goethite (Figure 2B), Ac:Pr was 0.67, close to the theoretical estimation of 0.5 according to the following stoichiometry (reaction 2; Stams et al., 1998).

(2) 3Glucose→4propionate+-2acetate+-2HCO3-+8H+

In those cultures, measured acetate and propionate accounted for consumption of about half of the glucose provided and presumably the remainder was consumed for cell carbon and/or directed toward ethanol, CO_2_, and CH_4_ which weren’t measured in that analysis. However, in the presence of goethite, Ac:Pr shifted dramatically in favor of acetate (2.42, Figure 2A), suggesting the following reaction (reaction 3; Stams et al., 1998) was the primary pathway for glucose fermentation in the presence of goethite and that goethite was an important hydrogen sink in the cultures, driving this shift in short chain fatty acid production.

3⁢Glucose+12⁢H⁢O2

(3)  →6acetate+-6HCO3-+12H++12H2

Theoretical hydrogen production from glucose fermentation via reaction 3 (after accounting for the acetate produced via reaction 2 based on measured propionate) is approximately 5× more than needed to reduce goethite to the extent that we observed in GluFeRB cultures (reaction 4). Other hydrogen sinks in the culture may include the Wood–Ljungdahl pathway of acetogenesis (Clostridiaceae, Supplementary Figure 2), which would also account for some of the measured acetate production, or methanogenesis by the *Methanobacterium* in the cultures.

(4) H+22FeOOH+4H→+2Fe+2+4HO2

Hydrogen as the electron donor for goethite reduction in culture GluFeRB has significant biotechnological implications where reduction of recalcitrant iron oxides is desired but providing hydrogen directly and/or on broad scale is not feasible (e.g., in stabilization of surface crusts during iron ore mine site remediation, Figure 1). Promoting microbial fermentation in mixed communities is an effective strategy to reduce crystalline iron oxides, in part because hydrogen produced from fermentation can be rapidly consumed with the abundant electron sink. Field-scale research to harness this process for reduction of iron ore mine waste in bioremediation approaches to re-cement iron duricrusts is underway.

## Data Availability Statement

The datasets generated for this study can be found in the GenBank Sequence Read Archive under BioProject accession number PRJNA561022.

## Author Contributions

EG undertook the field sampling, cultivation, chemical analyses, 16S rRNA gene analyses, searches for genes relating to Fe reduction, and metabolic reconstructions. JZ completed the bioinformatic analyses of the metagenome including genome assembly, binning, taxonomic classification, functional annotation, and searches for cytochromes and hydrogenases. GT provided the input regarding metagenomic analyses, metabolic reconstructions, and interpretation of culture data. GS undertook the field sampling and provided the input regarding cultivation and culture phenotype. All authors contributed to the manuscript preparation and editing.

## Conflict of Interest

The authors declare that the research was conducted in the absence of any commercial or financial relationships that could be construed as a potential conflict of interest.

## Footnotes

1 https://sourceforge.net/projects/bbmap/

2 https://github.com/wwood/CoverM

3 https://github.com/dparks1134/UniteM

4 https://github.com/Ecogenomics/GTDBTk

5 https://github.com/geronimp/enrichM

6 https://www.uniprot.org/proteomes/UP000000245
