# Supplementary material for: Goethite Reduction by a Neutrophilic Member of the Alphaproteobacterial Genus Telmatospirillum
Source: Front Microbiol. 2019 Dec 20;10:2938. doi: 10.3389/fmicb.2019.02938 (PMC6933298; doi:10.3389/fmicb.2019.02938)
Supplement: Supplementary file 1 [file Image_1.PDF]

OTU	Pool perched on canga	GluFeRB culture	% Identity to nearest named isolate in NCBI		
2	0.0	34.2	95.2%	*Elstera litoralis*	MH715222.1
1	0.0	33.2	98.0%	*Paludibacter jiangxiensis*	NR_146018.1
6	0.7	11.5	100.0%	*Methanobacterium lacus*	CP002551.1
3	0.3	9.6	100.0%	*Desulfovibrio desulfuricans*	AF098671 .1
4	0.0	4.6	99.2%	*Bacteroides xylanolyticus*	FR850058.1
5	0.0	2.9	99.6%	*Bacteroides xylanolyticus*	FR850058.1
8	10.7	0.0	99.2%	*Methanobacterium palustre*	EU293795
7	0.6	2.4	100.0%	*Clostridium aciditolerans*	NR_043557.1
9	4.3	0.0	82.9%	*Enterobacter nickellidurans*	AJ854063.3
10	4.1	0.0	98.8%	*Anaeromusa acidaminophila*	NR_024921.1
11	3.6	0.0	96.8%	*Desnuesiella massiliensis*	NR_14472
12	2.2	0.0	96.0%	*Raoultibacter timonensis*	NR_148574
14	2.0	0.0	100.0%	*Methanobrevibacter arboriphilus*	AY196663.1
16	1.6	0.0	97.2%	*Scytonematopsis maxima*	HQ012541.1
18	1.5	0.0	94.4%	*Ruminococcus bromii*	EU266549.1
19	1.5	0.0	100.0%	*Paraclostridium bifermentans*	MN294498.1
20	0.0	0.3	97.1%	*Paludibacter jiangxiensis*	NR_146018.1
21	1.2	0.0	99.6%	*Mycobacterium paragordonae*	CP025546.1
22	1.2	0.0	100.0%	*Curvibacter gracilis*	MN004843.1
23	1.1	0.0	99.2%	*Clostridium amylolyticum*	NR_044386.1
24	1.0	0.0	99.6%	*Planctomycetes* sp. Schlesner 638	X81951.1
25	1.0	0.0	96.0%	*Clostridium scatologenes*	CP009933.1
26	1.0	0.0	86.5%	*Meiothermus ruber*	AJ871173.1
27	0.9	0.0	99.2%	*Methanospirillum psychrodurum*	NR_133782.1
13	0.0	0.2	95.6%	*Elstera litoralis*	MH715222.1
28	0.8	0.0	99.2%	*Rhizobium wuzhouense*	MG857114.2
29	0.7	0.0	93.7%	*Choricystis parasitica* chloroplast	KM462878.1
30	0.7	0.0	96.8%	*Clostridium longisporum*	X76164.1
31	0.7	0.0	93.6%	*Planctomycetes* bacterium K2D	CP036350.1
32	0.7	0.0	100.0%	*Polynucleobacter victoriensis*	NR_159243.1
15	0.0	0.2	95.2%	*Elstera litoralis*	MH715222.1
34	0.6	0.0	97.9%	*Pelosinus propionicus*	NR_042513.1
35	0.6	0.0	98.8%	*Aquisphaera giovannonii*	CP042997.1
36	0.6	0.0	95.6%	*Raoultibacter timonensis*	NR_148574
37	0.6	0.0	92.9%	*Prolixibacter denitrificans*	NR_137212.1
38	0.6	0.0	96.0%	*Acetobacteroides hydrogenigenes*	NR_133950.1
39	0.6	0.0	98.0%	*Azonexus* sp. WE2	KU836931.1
17	0.0	0.1	98.7%	*Clostridium* sp. FAB1	KX242129.1
41	0.5	0.0	100.0%	*Polynucleobacter paneuropaeus*	CP030085.1
42	0.5	0.0	92.7%	*Dysgonomonas termitidis*	AB971823.1

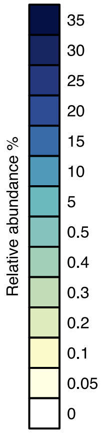

Supplementary figure 1: Heatmap analysis of 16S rRNA gene sequences from GluFeRB and the environmental sample from which it was enriched. Analysis was performed for OTUs clustered at a distance of ≤0.03. The scale bar indicates the relative abundance of each OTU within each sample, from white (least abundant) to blue (most abundant). The relative abundance of each OTU sequence is overlaid. The nearest named published isolate in the public domain and its accession number is given beside each OTU.
