# Supplementary material for: Goethite Reduction by a Neutrophilic Member of the Alphaproteobacterial Genus Telmatospirillum
Source: Front Microbiol. 2019 Dec 20;10:2938. doi: 10.3389/fmicb.2019.02938 (PMC6933298; doi:10.3389/fmicb.2019.02938)
Supplement: Supplementary file 2 [file Image_2.PDF]

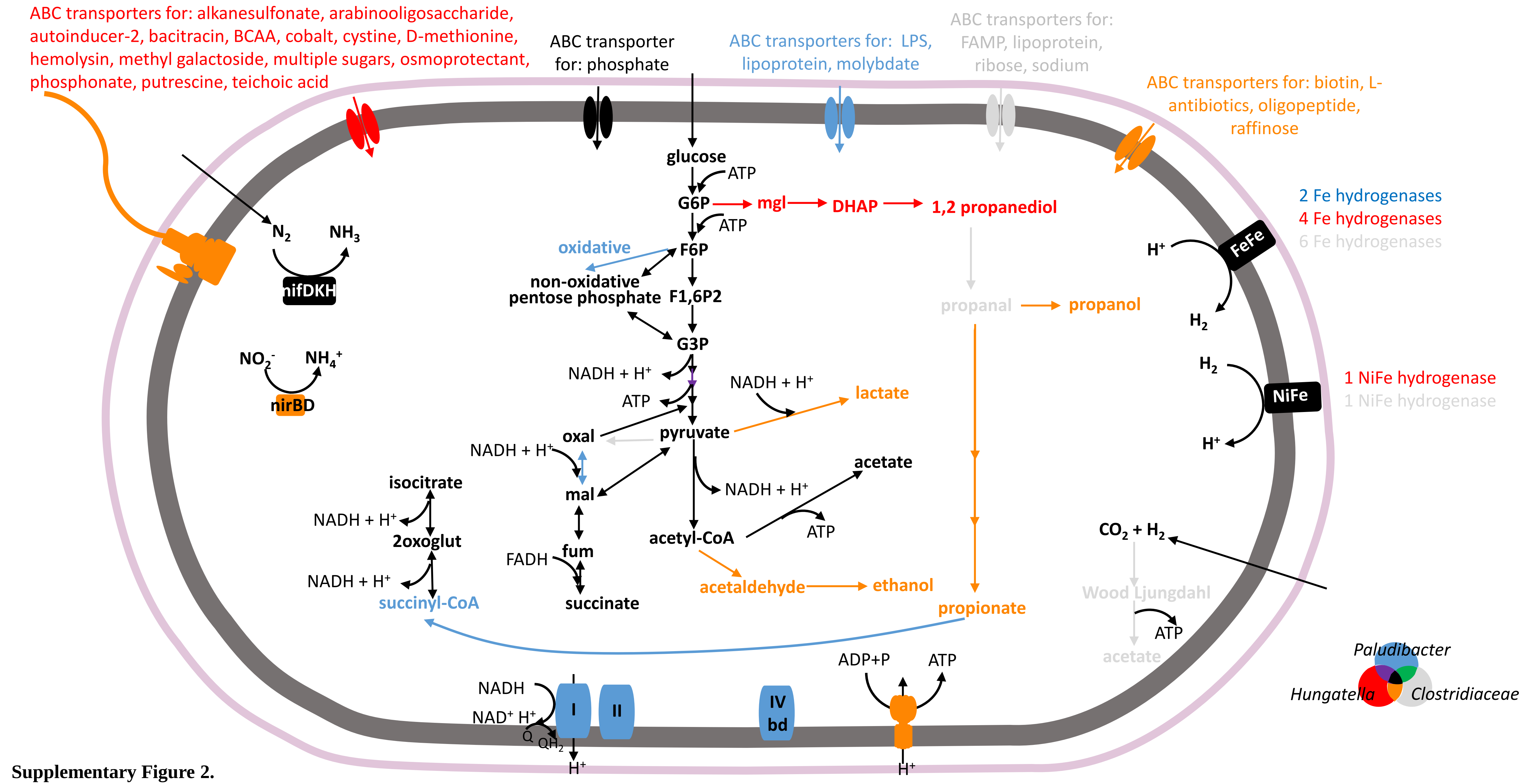

Supplementary Figure 2.
Metabolic reconstruction of the *Paludibacter* (blue), *Hungatella* (red), *Clostridiaceae* (grey) MAGs in culture GluFeRB. Pathways shared between these MAGs are represented by purple, green, orange or black, as indicated in the key. Abbreviations: branched chain amino acids (BCAA), N-formyl-4-amino-5-(aminomethyl)-2-methylpyrimidinelipopolysaccharide (FAMP), (LPS), glucose-6-phosphate (G6P), frustose-6-phosphate (F6P), fructose-1,6-bisphosphate (F16P2), glyceraldehyde-3-phosphate (G3P), oxaloacetate (oxal), malate (mal), fumarate (fum).
