# Supplementary material for: Goethite Reduction by a Neutrophilic Member of the Alphaproteobacterial Genus Telmatospirillum
Source: Front Microbiol. 2019 Dec 20;10:2938. doi: 10.3389/fmicb.2019.02938 (PMC6933298; doi:10.3389/fmicb.2019.02938)
Supplement: Supplementary file 4 [file Table_1.pdf]

Supplementary Table S1.
Deduced amino acid sequences of genes proposed to be involved in iron cycling in the *Telmatospirillum* MAG.

```
>SA7853BIN3_00738 Urocanate reductase
MSKTEVSRRSLLQAVTVGAVAVGGGAALNMAVPGTAEALVRKLPGNWDETWDVIVVGSGF
AGLAAAAESAAAGAKTILLEKMPSYGGNSIINGGVFAAWDDKWHYREKLNLGTDSPEQHF
QDILKGGDYYSIPELAKLVADGSADALAWVVDEGGAELRPTVTRAGGHTAYRTHAAKSGV
GREYTEALRKIAEKHGVKPTLGTEVTWIWRADADPKSPVLGVEVKRGKKVMNLKANKAVI
LASGGFSQNVDMRMSHNPRLTSDYNCTNHKGATGEIHKFAQAVGADTLQMNFIQLYPYAE
PETGLLDAQAVYPFNGPGYGILYVSKQGKRFVNELERRDVVAYAQINLGKDAKPTWSIFT
EAMILPMGGAPEEIEDGLKRGRFVKADTIPELAQKCKIPSDTLVDTVNKYIQYHKDGKDP
EFNKPITPAMFSLEKGPYYAIAQWPAVHFCMGGLRTNAKTQVIDVFGSPIPKLYAAGEVV
GGVHGSNRLGSNAIPTCIVFGRTAGRNAVLD
>SA7853BIN3_04245 Denitrification system component NirT
MNQSDEMVERRRPTLWGLAVGIWGFLARPSAQLSLGLLLLTGFLAGILFWGGFNWGLEMT
NNETFCRSCHEMDQNSYREYRTSVHDQNHAGVRATCPDCHVPHDWVHKMKRKIAASSEVF
HHLTGEIDTREKFEAKRLDLARSVWQTMKETDSRECRNCHSFDSMDLARQTDRARVAHTD
AETDGRTCIDCHKGIAHRLPAGMTEAERELNQSMPVGNRR
>SA7853BIN3_04711 Cytochrome c-552
MNPKVSVSSLLASVAFLALAAMPAQAIDWSGVQGKDVVLLYPGQASLEWVLTNGEHDGAA
KFKQGKNCKECHIGEENKMGPLAASGKVNEPQPIAGKPGTIPAKVKVAHDADTLYVHLEF
AEGTQPDAKQDAKYATKVSMMLSDGKVPEADRAGCWAACHDDSTDMASASGATRTKYLGK
PRAKITRPGGGDEMKSADELAKLKADGYGLEYWEAELNPGSPAHAAAYTVFDKREEAKPA
AVAAEASFANGTWSVTLSRKLHAGAPYKDIDPGKSFTVGFAIHAGHTARRFHYVSIDQSL
VLDQGTADFVAAKK
>SA7853BIN3_04712 hypothetical protein
MIFRKLVIGLVSGLAITLSSAAARAAEDPAPATTPAVYAEKGDQTCLKCHDNEQVTSILK
TPHGTKGDIRTPAANHGCESCHGASPEHVASRPAKGEKKALPAIRFSGPNISPVADRNAV
CLTCHENSARINWQGSQHASNDVACSTCHTVHAQKDPVLVKATQPDKCFTCHAQQRAESF
QRSHHPVREGKVSCADCHNVHGSPGPKLVKEVSINETCYNCHAEKRGPMLWEHQPVRENC
DNCHTPHGSSQAALLTERMPYLCSSCHSATGNNSGGWFGGSRSLPGHSPSNAVMNNELNN
RSCLNCHSNIHGSNSPGGQAFLR
>SA7853BIN3_04713 hypothetical protein
MKKQYSLRFLALAGVCLAPLAAQAQDFDLGDAPQAAAPAKQYTNEIDVGVRYQSSTSPLY
GRYTGTDSKGFGSLGGFHLESTMTPASGAPLKVEATGTNLNFQPDHQGPNNALAPESEVN
VSAGQQGIWKAGAYYNAITYTGQKFLTPYTASGNLAPGQQAFGGQTITGYTAAGVPILSA
VPAAGKPGTAAYYVNNAFPEFQTTAGTRRDIGGVDGKYIIDAWTITTGFRHEHKEGTTLQ
TMYTSNAGIAFPQPVNYDTDRYNVTAAFNTRRLQAQLGYNFSKFSDNASFFTSPYLFATS
ATAQSVSVYSQPPSNFAHYVNGAAGYNLTPTTRITSNFQYGLEMSDGALGAGTATPLSEI
GGAASAARLALNPGGDQMARVYNANLGVTSRPMAGLDVKVGYGIDGRENSSSPMTVYGNS
HGDGAPALIGNILQQNWTKQKATLEAGYKVLPNTKVSVGYRLDDVHRSAGAVASLPMSSL
GWVGHSTENTEWIKVSDHSLAQLDSSVTYEHAVRTGLMELAPGSGTVTNGQVQNSSMPFY
EAPRTSDRVKLRADYMPADQWTIGANARYEANHYNYSSTITGTQRDYNTSAGPDITYSPT
KAISLHGFYTYEEIYYVNRGNGVPSTLNKNYGWSAATTDSVHTAGVSADWQVSERLKVGA
EYTFSYGDIGYNLYDGGLALNTATAASYYNVSNLPTIDSSMHSVKLRAEYKLTDAITLMG
GYGFDMYKDNDWSYGWSPGMASNASGLAAPLGVNTFTXXXGSPDFCPLGRSASSGPFFFT
LAGAQAPALFSSPWRERKLRPLFFLHSARHRRPA
>SA7853BIN3_04795 Citrate synthase
MTQDENKDLVTLVDGASGQRFELPLIPGTVGPKVIDIRSLYGLTGYFTYDPGYTSTGSCE
SKITYIDGDNGILLHRGYPIDQLAEHSDYLEVCYLLLRGELPDAKQKAKFEGDIRNHTMV
HEQINSFYRGFRRDAHPMAVMCGVVGALSAFYHDSLDIADPHQRMVASHRMIAKMPTIAA
WAYKYSRGEPFIYPRNSLSFAENFLHMMFATPCEEYKVNPVLARAMERILILHADHEQNA
STSTVRMAGSSGANPFACIAAGIASLWGPAHGGANEAVLQMLTQIGSKERIPEFIARAKD
KNDPFRLMGFGHRVYKNYDPRAKVMQRTCHEVLDELGVRDEPLLELAMELERIALEDPYF
VDKKLYPNVDFYSGIIFKAMGIPVSMFTVLFAVARTVGWVAQWNEMIGDPHQKIGRPRQL
YTGAAHRDFVPLHARG
>SA7853BIN3_04796 hypothetical protein
```

MRTGVPMPDFDAWDIIKVPFPYTDRPVREHRPAVVVAANGIQREHGLLWVLMITSAENRG
WPGDVGVSNLSEAGLPAESVVRTAKIATIEIKEAERIGSLPADDRILVAHYLTTGLARTT
N
