# Supplementary material for: Goethite Reduction by a Neutrophilic Member of the Alphaproteobacterial Genus Telmatospirillum
Source: Front Microbiol. 2019 Dec 20;10:2938. doi: 10.3389/fmicb.2019.02938 (PMC6933298; doi:10.3389/fmicb.2019.02938)
Supplement: Supplementary file 3 [file Image_3.PDF]

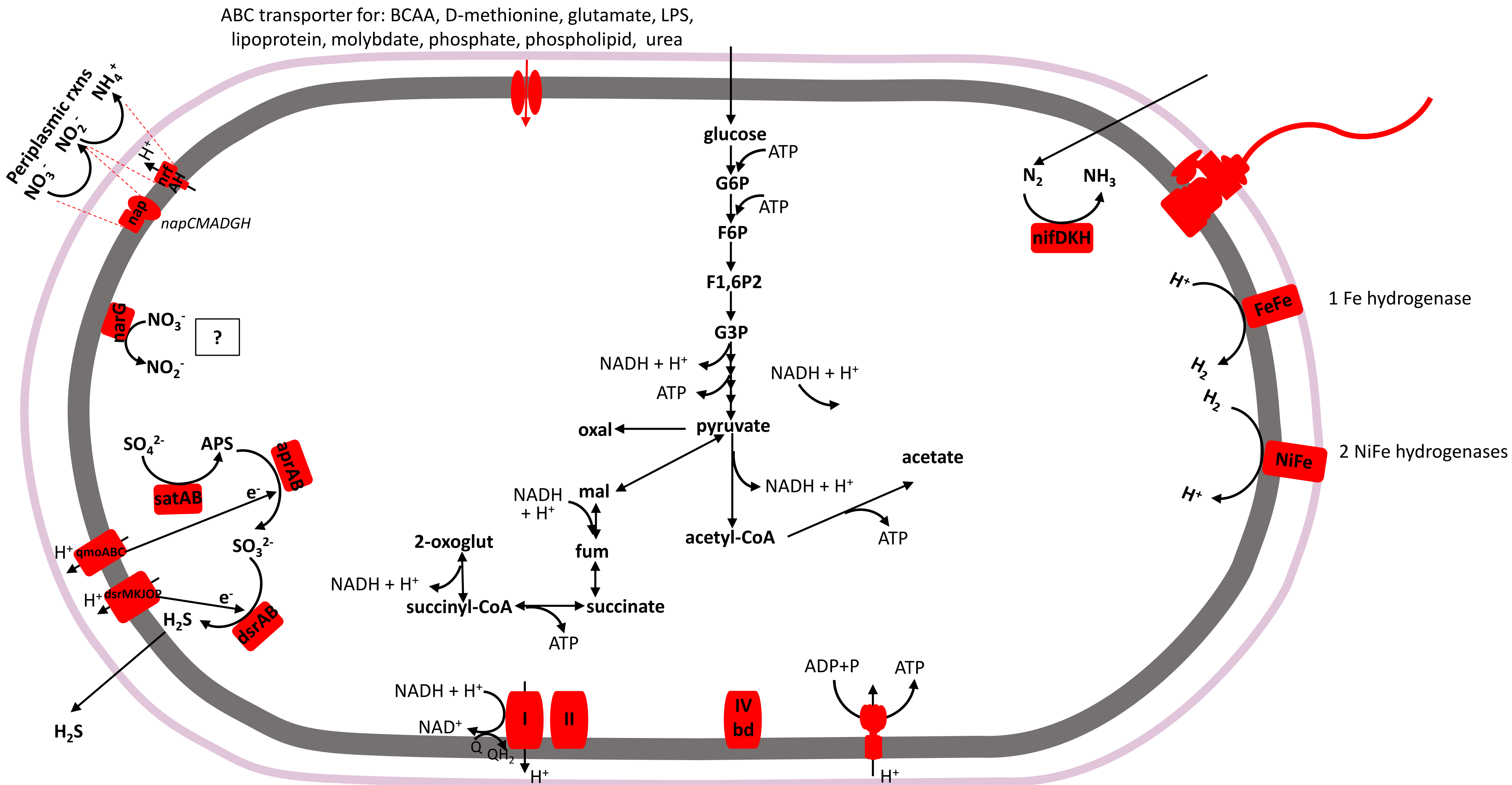

Supplementary Figure 3.
Metabolic reconstruction of the *Desulfovibrio* MAG in culture GluFeRB. Abbreviations: branched chain amino acids (BCAA), (LPS), glucose-6-phosphate (G6P), frustose-6-phosphate (F6P), fructose-1,6-bisphosphate (F16P2), glyceraldehyde-3-phosphate (G3P), oxaloacetate (oxal), malate (mal), fumarate (fum), oxoglutarate (oxoglut), adenosine-5'-phosphosulfate (APS). The boxed question mark denotes the fact that only the catalytic subunit of the complex required to perform this reaction is present.
